# Treatment of Canine Disc-Associated Cervical Spondylomyelopathy with a Cervical Distraction–Stabilization Technique (C-LOX Combined with LCP Plate) and Clinical Outcomes

**DOI:** 10.3390/ani13162549

**Published:** 2023-08-08

**Authors:** Marco Tabbì, Giuseppe Barillaro, Claudia Dina Interlandi, Simona Di Pietro, Domenico Fugazzotto, Giovanna Lucrezia Costa, Nicola Maria Iannelli, Daniele Macrì, Vincenzo Ferrantelli, Francesco Macrì

**Affiliations:** 1 Department of Veterinary Sciences, University of Messina, 98168 Messina, ME, Italy; marco.tabbi@unime.it (M.T.); dipietros@unime.it (S.D.P.); giovannalucrezia.costa@unime.it (G.L.C.); nicolamaria.iannelli@unime.it (N.M.I.); fmacri@unime.it (F.M.); 2 CVSG (Clinica Veterinaria San Giorgio), Via Vecchia Pentimele, 63, 89121 Reggio Calabria, RC, Italy; info@cvsg.it; 3 Ospedale Veterinario San Francesco Trevisovet s.r.l., Strada Feltrina 29, 31038 Castagnole, TV, Italy; domenicofugazzotto@gmail.com; 4 Institute Zooprofilattico Sperimentale of Sicily, Via G. Marinuzzi, 3, 90129 Palermo, PA, Italy; daniele.macri@izssicilia.it (D.M.); vincenzo.ferrantelli@izssicilia.it (V.F.)

**Keywords:** cervical spondylomyelopathy, neurosurgery, dog, distraction–stabilization technique, anchored fusion device, LCP plate

## Abstract

**Simple Summary:**

The aim of the study was to evaluate the outcomes of a cervical distraction–stabilization technique using an intervertebral anchored fusion device (C-LOX) combined with a locking compression plate (LCP plate) for the treatment of disc-associated cervical spondylomyelopathy (DA-CSM) in dogs, based on clinical and radiographical follow-up data. Thirteen dogs affected by DA-CSM were included in the study. After the surgical procedure, an improvement in neurological status was documented in 9/13 cases. This cervical distraction–stabilization technique seems to be a valuable surgical alternative to treat this canine pathology.

**Abstract:**

Canine disc-associated cervical spondylomyelopathy (DA-CSM) is a form of caudal CSM, characterized by the compression of the spinal cord and nerve roots due to an intervertebral disc protrusion. It is more frequent in large canine breeds. A variety of surgical techniques has been proposed for DA-CSM. The aim of the study was to evaluate the outcomes of a cervical distraction–stabilization technique using an intervertebral anchored fusion device (C-LOX) combined with a locking compression plate (LCP plate) for the treatment of DA-CSM in dogs, based on clinical and radiographical follow-up data. Thirteen dogs affected by DA-CSM were included in the study. After the surgical procedure, an improvement in neurological status was documented in 9/13 cases. This cervical distraction–stabilization technique seems to be a valuable surgical alternative to treat this canine pathology.

## 1. Introduction

Caudal cervical spondylomyelopathy (CCSM), also known as wobbler syndrome, is a multifactorial disease affecting large and giant breed dogs that causes compression of the spinal cord and nerve roots [1,2,3,4,5,6,7,8,9].

This compression, caused by a combination of several factors such as degenerative and progressive disorders of the intervertebral disc, ligaments, and articular facet joints, leads to varying degrees of neurological dysfunction, cervical hyperesthesia, and pain [1,2,10,11] There are two recognized forms of CSM, which can occur separately or concurrently: disc-associated cervical spondylomyelopathy (DA-CSM) and osseous-associated cervical spondylomyelopathy (OA-CSM) [1,10,11,12,13,14,15]. Compression of the spinal cord due to DA-CSM typically occurs at the intervertebral disc spaces between the fifth (C5) and seventh cervical (C7) vertebrae in large breeds of dog such as Dobermann Pinschers [16]. Giant breeds of dog such as Great Danes also develop lesions of the more cranial vertebrae, including stenosis of both osseous and soft tissue origin [17]. This has also been observed in chondrodystrophic breeds such as Basset Hounds [18]. 

Various surgical approaches for the treatment of DA-CSM have been described. The proposed surgical techniques are commonly divided into direct decompressive (by a ventral or dorsal approach), indirect decompressive with motion preservation (total disc replacement by arthroplasty), and indirect decompressive without preservation of motion [19]. The latter involves vertebral distraction and stabilization in order to restore the intervertebral disc and foraminal width, and to stabilize the vertebral segment to prevent ongoing dynamic concussive injury to the spinal cord [10,11,20,21]. The most commonly decompressive procedures include fixation methods with or without the implantation of intervertebral spacers [22,23,24], alone or in combination with other devices, such as screws or ventrally applied locking plates [25,26,27,28,29,30,31,32,33,34]. Among the intervertebral devices described in the literature, C-LOX combines the effect of an intervertebral fusion cage with a ventral locking plate in a single implant. The features of C-LOX allow its easy and safe placement, decreasing the risk of iatrogenic injury by spinal cord penetration thanks to monocortical locking screws [22]. The main advantage of the additional ventral stabilization compared with other intervertebral devices is the reduced risk of ventral displacement with implant failure [22,35]. Despite this, the rate of complications such as loosening or breakage of the screws resulting in ventral displacement of the implant remains high [22,23]. Combining an interbody fusion cage with plate fixation has been found to reduce the displacement and subsidence of the implant, increase the construct’s rigidity [36], and increase the rate of spinal fusion in humans [37].

Considering the variety of treatments proposed for this pathology and the complications associated with implants, the aim of this study was to describe the surgical treatment of canine traction-responsive DA-CSM through a cervical distraction–stabilization technique using a spinal disk replacement implant associated with a ventral fixation plate, and to report the outcomes and complications in 13 dogs.

## 2. Materials and Methods

### 2.1. Inclusion Criteria

Between 2014 and 2018, 19 dogs were treated for traction-responsive DA-CSM. The patients included in this study underwent a surgical procedure of cervical distraction–stabilization using an intervertebral cage (C-LOX, Rita Leibinger Medical^®^, Muehlheim, BW, Germany) combined with a ventral plate (locking compression plate (LCP), De Puy Synthes^®^, West Chester, PA, USA). 

Dogs affected by systemic diseases (*n* = 6) were excluded from the study, as well as subjects who failed to comply with the follow-ups planned after the surgical procedure (*n* = 20). 

Thirteen client-owned dogs met the inclusion criteria. For each patient, informed consent was obtained.

The definite diagnosis of DA-CSM was confirmed by magnetic resonance imaging (MRI) using a Hitachi Airis Vento 0.3 Tesla machine (Hitachi Medical Corporation, Tokyo, Japan) which also allowed for surgical planning and implant selection. All dogs were sedated and placed in dorsal recumbency. The protocol of the MR examination included T2-weighted series (T2W; TR 4000 ms; TE 120 ms) in the sagittal and transverse planes and T1-weighted (T1W; TR 520 ms; TE 20 ms) pre- and postcontrast series in two transverse planes for intervertebral disc regions. Then T2-weighted images in sagittal and transverse planes were taken under traction of the patient’s neck to evaluate the dynamism of the cervical lesion.

Each dog underwent clinical, neurological, and radiographical examinations before and immediately after the surgical treatment and at 6 weeks after surgery (short-term follow-up). Radiographical examinations were performed under sedation to obtain optimal patient positioning. Clinical and neurological assessments were also performed at 6 months after surgery (medium-term follow-up). Biochemical analysis (complete blood count, azotemia, urea, transaminases, total proteins) was performed preoperatively.

Short-term neurological status was compared pre- and postoperatively, and dogs were rated as normal, improved with residual dysfunction, unchanged, or worsened. Neurological signs were graded according to a modified Frankel scale [38]: Grade 0, normal neurological examination; Grade 1, cervical pain during manipulation; Grade 2, mild pelvic limb ataxia with no proprioceptive deficits; Grade 3, mild ambulatory tetraparesis with proprioceptive deficits; Grade 4, severe ambulatory tetraparesis with proprioceptive deficits; Grade 5, non-ambulatory tetraparesis; Grade 6: tetraplegia.

Postoperative X-rays were performed in both orthogonal standard projections to assess the implant’s placement, stability, and integrity. Ventral vertebral fusion (non-interrupted new bone formation) was assessed at the short-term follow-up by radiographs.

Complications and relapses of the clinical signs were recorded. Complications were classified as major or minor, and whether or not they required additional treatment to resolve, respectively; an unacceptable permanent function or death were considered to be catastrophic complications. Moreover, complications were divided into intraoperative, short-term (6 weeks after surgery), and medium-term (6 months after surgery).

### 2.2. Surgical Technique

The anesthetic protocol included premedication with acepromazine (Prequillan, Fatro S.p.A., Bologna, Italy; 0.02–0.04 mg/kg) and methadone (Semfortan 10 mg/mL, Eurovet Animal Health B.V., Bladel, The Netherlands; 0.2 mg/kg IM), the induction of anesthesia with propofol (Propovet Multidose 10 mg/mL, Zoetis S.r.l., Rome, Italy; 4 mg/kg EV), and maintenance with sevoflurane (SevoFlo 100% 250 mL, Zoetis Belgium SA, Louvain-la-Neuve, Belgium; 3.5%). Intraoperative pain management with fentanyl (Fentadon, Eurovet Animal Health B.V., Bladel, The Netherlands; 2–4 mg/kg IV) at a constant rate of infusion was performed. During anesthesia, electrocardiography, heart and respiratory rate, end-tidal CO_2_, saturation of blood O_2_, and non-invasive blood pressure were recorded.

All dogs received antibiotic treatment with cefazolin (Cefazolina TEVA, Milan, Italy; 20 mg/kg) administered IV at the time of the induction of general anesthesia and then every 90 min until the end of the procedure. 

After the induction of general anesthesia, each patient was positioned in dorsal recumbency with the caudal cervical spine raised about 5 cm above the table by a padded support, avoiding excessive extension. The thoracic limbs were caudally distracted and fixed to both sides of the chest. The maxilla was fixed to the operating table to maintain axial alignment and prevent movement during surgery. A standard ventral median approach to the cervical spine was performed [39]. A median longitudinal incision of the skin and subcutis was made, moving the various planes up to the muscular one. The sternohyoid and sternocephalic muscles were longitudinally incised to isolate the trachea and esophagus, which were moved laterally through the use of a retractor (e.g., a Balfour retractor), together with the large vessels and the recurrent nerves.

The intervertebral space involved was first localized through palpation of the ventral processes and subsequently exposed by incision of the longissimus colli muscle with the aid of a bipolar electrosurgical unit for hemostasis and a periosteal elevator. The vertebrae were carefully distracted by a Caspar retractor.

**Figure 1:**
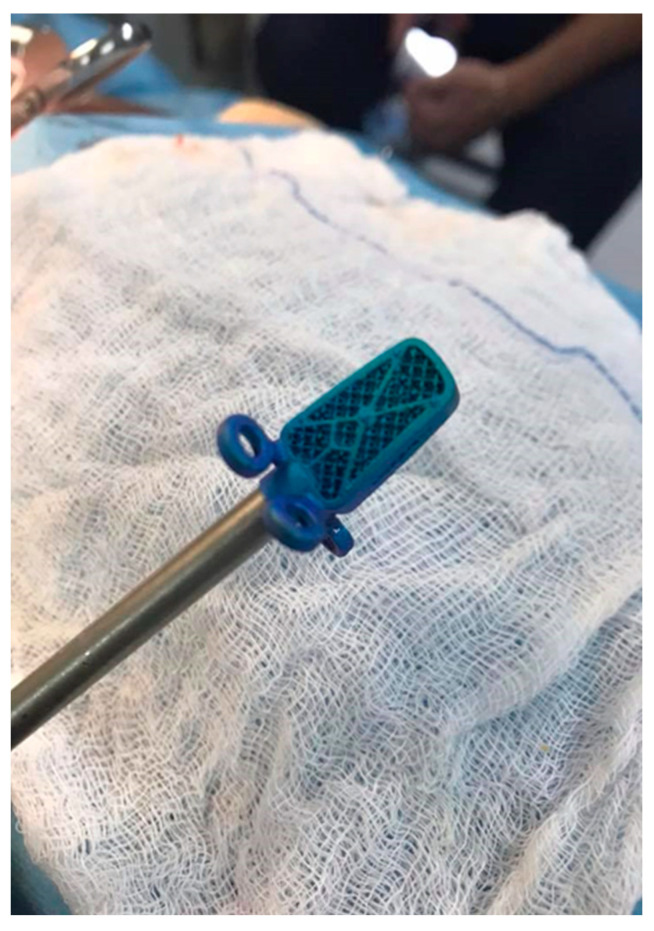
Titanium cage (C-LOX, Rita Leibinger^®^) used in the surgical technique: intraoperative photo.

**Figure 2:**
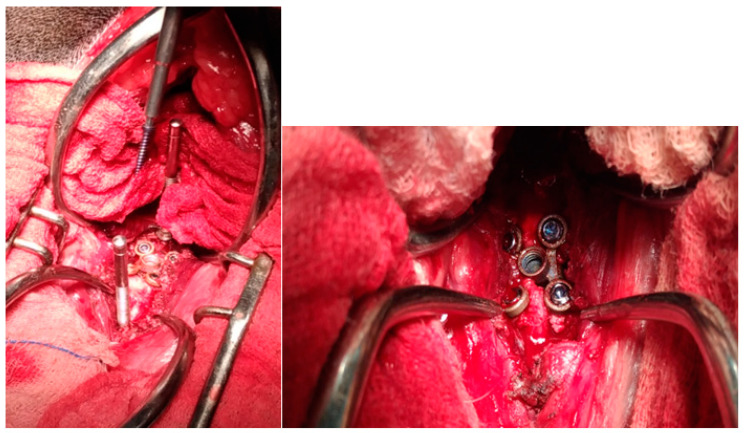
Application of the titanium cage (C-LOX, Rita Leibinger^®^): intraoperative photo.

The intervertebral disc was incised and, with the help of a spinal disc broaching curette, all the remains of the nucleus pulposus were carefully removed from the intervertebral space, safeguarding the subchondral bone and the dorsal part of the fibrous ring. After the endplate chondrectomy had been performed, the correct fit was verified by inserting the test spacer, fixed to the special positioning rod. Once the optimal size was determined, the trial spacer was removed and replaced by the corresponding C-LOX device (Rita Leibinger GmbH & Co. KG, Mühlheim/Donau, Germany) (Figure 1 and Figure 2). The distractor was removed, allowing for vertebral repositioning and, if necessary, the “ears” were slightly bent for optimal bone contact. Implants of different sizes, with variations in height (14 mm, 16 mm, 18 mm, 20 mm), thickness (4 mm, 5 mm, 6 mm, 7 mm, 8 mm), and width (8 mm, 10 mm, 12 mm) were used. Two caudal and two cranial 1.8 mm locking screws were incorporated into the plate by the means of slightly flexible “ears” featuring threaded, locking screw holes. The 1.8 mm screws were of several lengths, including 10 mm, 12 mm, 14 mm, 16 mm, 18 mm, 20 mm, 22 mm, and 24 mm.

**Figure 3:**
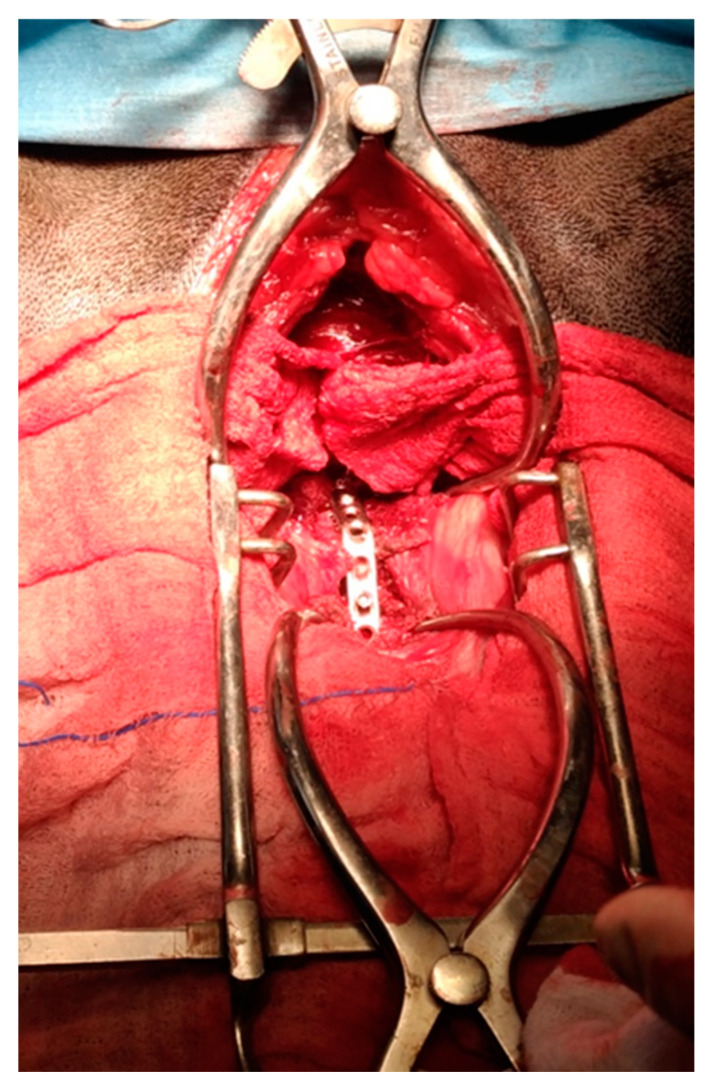
Application of the locking compression plate (Synthes^®^).

The threaded drill guide was attached to the implant, and a dedicated 1.8 mm drill bit was used to drill holes for the locking, self-drilling cortex screws. This tip forms a unit with the screw’s centering sleeve, limiting the penetration to the ventral cortex and eliminating the risk of iatrogenic medullary damage. The four screws were then inserted into the appropriate holes, concluding the anchoring of the implant to the vertebral bodies. An autologous cancellous bone graft (ACBG) was then removed from the proximal humerus and placed along the ventral surface of the disc space and the adjacent vertebral bodies. Finally, to ensure the greater stability of the implant, the ventral fixation plate (LCP plate, locking compression plate, Synthes^®^) was applied (Figure 3). Paired 7-hole (2.7 mm, 67 mm in length) LCP plates and monocortical locking screws were utilized for all patients. The plates were aligned on the underlying vertebrae, and three screws on each vertebral body were placed in the respective holes. This left only one open hole above the C-LOX device.

The procedure was closed routinely. The mean duration of surgery was 1.9 h (range: 1.5–2.6 h).

During the first 36 h postoperatively, each dog received IV cefazolin twice daily. Postoperative care and analgesia were adapted to the neurological status and clinical course of the individual patient. For analgesia, methadone at 0.2 mg/kg intramuscularly was administered as needed, and no later than 5 days.

Anti-inflammatory drugs (prednisolone, Vetsolone, PROVET S.A., Bayer, Milan, Italy; 0.5–1 mg/kg) were orally administered for 10–15 days postoperatively; antibiotic (cefadroxil, Cefa-Cure Tabs cpr, MSD Animal Health S.r.l., Milan, Italy; 20 mg/kg SID *os*) and gastroprotective treatments (omeprazole, 1 mg/kg and sucralfate (50 mg/kg) were applied for 10 and 20 days, respectively. Finally, oral supplementation with palmitoylethanolamide (PEA) and quercetin (Alevica^®^, Innovet, Saccolongo—Padua, Italy) SID was prescribed for at least 20 days.

All patients underwent physiotherapy sessions to accelerate motor rehabilitation, as previously reported [34]. The owners were given clear and strict instructions to follow during the postoperative period. These include maintaining strict confinement of the patient in a confined space, avoiding sudden exertion or jumps from any height, and providing as few walks as possible, of short duration and always with a support harness. 

At 6 weeks, the brace was removed, and control cervical radiographs were performed. If the radiographic follow-up showed positive results, a gradual increase in walking frequency and length was allowed for another 6 weeks before resuming unrestricted activity.

### 2.3. Statistical Analysis

The calculation software Prism 9.0 (Graph Pad Software, San Diego, CA, USA) was used to perform the statistical analysis. A 2-tailed, paired Student’s *t*-test was used to compare the preoperative neurologic scores with postoperative and 6-week and 6-month outcomes. Significance was established for all tests at *p* < 0.05. 

## 3. Results

### 3.1. Demographics

The study group included 13 dogs with ages ranging from 6 to 10 years, and nine males (70%) and four females (30%) (Table 1). The weight ranged from 27 kg to 63 kg, with a mean value of 40 ± 11 kg). Dogs were fed with commercial food and led both indoor and outdoor lifestyles. 

### 3.2. Preoperative Assessment

Diagnosis was achieved with radiographs and MRI. 

**Figure 4:**
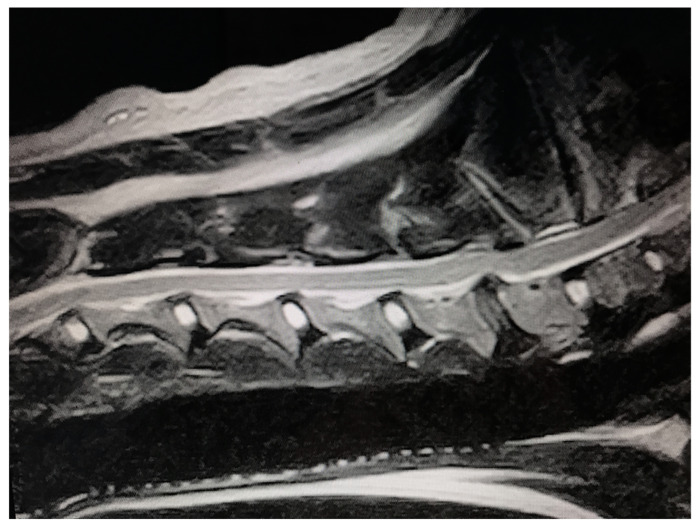
Sagittal T2-weighted MR image of a Swiss Shepherd (male, 94 months).

The MRI examination showed that the main lesion was located at C5–C6 in 10 (77%) dogs and at C6–C7 in 3 (33%) dogs (Figure 4).

At the level of C7, moderate vertebral tipping was observed. At the level of C6–C7, a bulging intervertebral disc was also noted, with a reduction in the diameter of the dorsal–ventral canal at the space affected.

The most frequently observed clinical signs were hindlimb weakness that started months before and gradually worsened, ambulatory tetraparesis with mild neurological deficits, and, in one case, non-ambulatory tetraparesis. Hindlimb manifestations were more severe than forelimb ones in all patients. On clinical examination, all patients showed marked atrophy of the supra- and infraspinatus muscles, which made the spine of the scapula easily palpable. 

Due to proprioceptive deficits, the skin on the back of the hind and front legs exhibited abrasions caused by continuous rubbing against the ground. No consistent abnormalities were found in the complete blood counts or the serum’s biochemical profiles.

On neurological examination, 9/13 dogs (70%) showed a posture with a lower neck, 12/13 dogs (93%) showed ambulatory tetraparesis with a short and stiff gait of the forelimbs and an dysmetrical gait of the hindlimbs (two-neuron gait), proprioceptive deficits in all limbs, hyporeflexia of the flexor reflex of the hindlimbs, and normal spinal reflexes (flexor and patellar) in the hindlimbs. One dog (1/13, 7%) showed non-ambulatory tetraparesis (Dog 7). All patients showed normal cranial nerves. Neck pain was often present.

### 3.3. Postoperative Assessment

**Figure 5:**
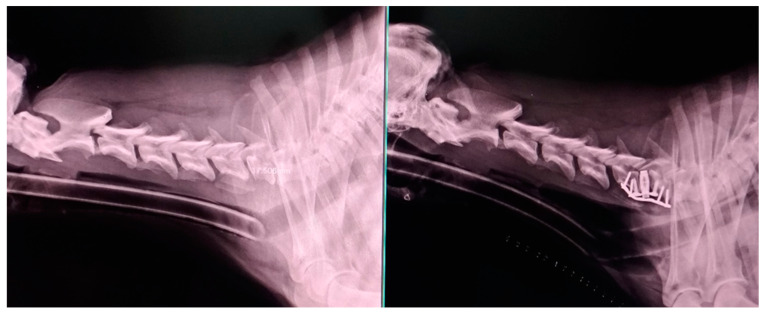
Radiographic images of a Swiss Shepherd (male, 94 months): lateral view of the cervical spine before and after surgical treatment. The correct application of the titanium cage and plate can be observed.

Figure 5 shows the radiographic study performed before and after the surgical procedure in a treated dog.

In one dog (Dog 13), detachment of the fixation plate occurred within some days of surgery. A second surgery was performed to remove the plate and replace it with a new one. No complications occurred during the replacement procedure. The new plate was the same as the others. 

The neurologic score of four dogs was unchanged immediately postoperatively, and improved in nine dogs.

The neurologic scores at 6 weeks after surgery were significantly different from the preoperative scores (paired *t*-test, *p* = 0.0003; *t* = 5.017, df = 12). The neurologic score of 9/13 dogs (70%) were improved at 6 weeks postoperatively. Five dogs showed cervical pain with or without mild to moderate pelvic limb ataxia. A neurologic score of 0 was noted in 4/13 dogs at 6 weeks postoperatively. Out of all cases, 3/13 (23%) dogs had no neurological improvement, and 1/13 (7%) had a worsening of the symptoms (Dog 7), deteriorating from severe non-ambulatory tetraparesis preoperatively to tetraplegia immediately postoperatively. 

At the medium-term examination, 10 dogs were neurologically normal without signs of pain. One dog had mild hindlimb ataxia without signs of pain. A significant difference between the neurologic scores at 6 months and 6 weeks postoperatively was reported (*p* = 0.0028; *t* = 3.742, df = 12).

**Figure 6:**
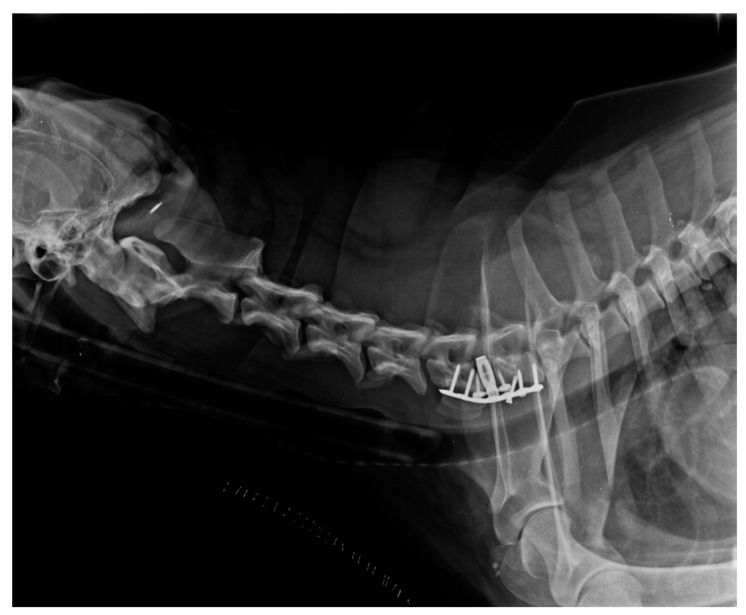
Short-term radiographic image of a treated dog: the loosening of a screw can be observed.

No vertebral fusion occurred in all dogs at medium-term follow-up. Minor complications were seen in only one dog, showing the loosening of a screw, which was not associated with neurological deterioration (Figure 6).

No complications that caused unsolvable permanent damage or for which euthanasia was required were observed.

## 4. Discussion

Caudal cervical spondylomyelopathy (CCSM) is a canine progressive disease characterized by degenerative changes that, through direct compression of the spinal cord and the surrounding vascular and nerve structures, can potentially lead to severe neurological damage and permanent disability [40].

Identifying the early signs and providing an early diagnosis and effective treatment before the development of irreversible spinal cord damage are essential to maintaining canine quality of life.

DA-CSM, frequently identified in large-breed dogs [1], can cause non-traction-responsive (static lesions) and traction-responsive (dynamic lesions) cord compressions [41]. The ideal management for traction-responsive DA-CSM is still controversial. Conservative treatment, despite initial improvement, may lead to further progression of the clinical signs [6,14,41].

When a dynamic lesion has to be managed, the surgical procedure should have the purpose of reducing neurological deficits and relieving the compression of the spinal cord, stabilizing the cervical vertebrae [1,41].

The surgical techniques proposed for the treatment of DA-CSM in dogs reported positive results in 70–90% of DA-CSM cases [5,16,22,24,25,27,28,29,30,32,33,34].

Recently, the use of intervertebral body cages with or without adjuvant ventral plates to realize an interbody arthrodesis has become a more popular technique for dogs [24,27,28,29,30,32,33,34,40].

In the choice of a surgical technique for the treatment of DA-CSM, the potential risks of complications such as implant failure and subsidence should be considered [16,22,24,27,28,29,30,31,32,33,41]. Iatrogenic damage is only one of the several potential risks of distraction–fusion surgery, along with postoperative neurological deterioration, penetration of the implants into the neural tissue, subsidence of the implant, and adjacent segment disease (ASD or domino lesions). Early implant failure with loss of distraction before stabilization is another very common complication [25,30,42]. Intervertebral cages also have a high risk of ventral dislocation, and further stabilization may be required, using monocortical screws [27,43] or spinal locking plate systems, which are widely used in human medicine [11].

The C-LOX spacer used in this study combines an intervertebral spacer with a conventional screw fixation mechanism, and it was designed to stabilize and restore the function of the cervical spine. Despite fixation of the implant with monocortical screws, a recent study evaluating its efficacy in 37 dogs reported screws loosening as the most common complication, making revision surgeries with a ventral plate necessary. The screws alone may not have been able to withstand the high torsional forces occurring in this region of the cervical spine, mostly concentrated at the junction between the head and the shaft, resulting in loosening or breakage of the screws and ventral dislocation of the implant [22].

In our study, to minimize the rate of complications of the C-LOX device used alone, such as screw loosening, breakage, and ventral cage dislocation, and to ensure the increased rigidity and stability of the implant, we combined this intervertebral device with a ventral fixation plate system.

Combining a fixation plate with a distraction device as a cage has been found to improve the rate of intervertebral fusion in humans [37], with the rate of the non-union of bone grafts being up to 26% without the use of a cervical plate [44]; moreover, locking plate fixation has become a widely accepted method in the treatment of DA-CSM [22].

In our study, vertebral body fusion was not evident in any dog at medium-term follow-up, in accordance with previous studies reporting that serial radiographs over a longer period may be required to accurately assess bone bridging [23,32].

The effects of this combination of an intervertebral spacer and a fixation system with ventral plates have already been documented in two previous studies, showing both short- and long-term positive clinical and radiographical outcomes [24,34]. Despite some complications, such as loosening of the implant, plate fixation promotes better stability, ensuring good construct rigidity [34].

Based on our findings, the use of C-LOX device in combination with a ventral locking compression plate may be a viable surgical treatment for DA-CSM. The surgical procedure was relatively simple, the duration was considered to be reasonable, and the duration of hospitalization was comparable with other techniques. No substantial intraoperative complications were detected, and only one case had a displacement of the angular stability plate. Nine dogs (70%) had neurological improvements 6 months after surgery.

Our study presents some limitations, such as the lack of a control group and the small number of dogs enrolled. Further studies are needed to confirm the efficacy of this methodology in a larger sample, although the enrollment of treated dogs could be made difficult by the high costs of the procedures.

Moreover, a radiological assessment at medium-term follow-up in order to evaluate the status of the implant and fusion was not performed due to a lack of permission from the owners to sedate their dogs again. This condition may have underestimated both the degree of improvement and the incidence of implant complications, although clinical and neurological evaluations of the treated dogs showed significant neurological improvements and the absence of clinical signs.

## 5. Conclusions

The clinical outcomes of the 13 dogs reported here were good. The application of the C-LOX device in association with fixation plates and ACBG led to satisfactory results for the treatment of DA-CCSM, as demonstrated by the clinical and radiographic findings. Our results provide useful information to help surgeons broaden their choices of surgical techniques for the treatment of DA-CCSM.

## Figures and Tables

**Table 1:** Signalment and neurological scores in 13 dogs with CCSM.

Dog	Breed	BW (kg)	Sex	Age (y)	Neurological Score (MFS)			
					Presurg.	Immediate Postsurg.	Short-Term Follow-up	Medium-Term Follow-up
1	Dobermann	35	F	6	3	2	0	0
2	Dalmatian	27	F	9	4	3	2	0
3	Weimaraner	40	M	9	3	2	0	0
4	Dobermann	45	M	8	3	2	0	0
5	Dalmatian	32	M	8	4	4	3	1
6	Dobermann	42	M	7	4	3	0	0
7	Bernese Mountain Dog	63	M	6	5	5	6	6
8	Dalmatian	30	M	10	4	3	3	0
9	Dobermann	34	F	7	3	2	2	0
10	Swiss Shepherd	62	M	6	4	3	1	0
11	Dobermann	32	F	7	4	3	1	0
12	Labrador Retriever	35	M	8	3	2	2	0
13	Dobermann	44	M	7	3	3	2	1

Abbreviations: BW, body weight; MFS, Modified Frankel Scale; F, female; M, male; y, years; Presurg., pre-surgery; Postsurg., post-surgery.

## Data Availability

The data presented in this study are available on request from the corresponding author.
